# Substrate-Flexible Two-Stage Fed-Batch Cultivations for the Production of the PHA Copolymer P(HB-*co*-HHx) With *Cupriavidus necator* Re2058/pCB113

**DOI:** 10.3389/fbioe.2021.623890

**Published:** 2021-03-22

**Authors:** Lara Santolin, Saskia Waldburger, Peter Neubauer, Sebastian L. Riedel

**Affiliations:** Chair of Bioprocess Engineering, Institute of Biotechnology, Technische Universität Berlin, Berlin, Germany

**Keywords:** *Cupriavidus necator*, *Ralstonia eutropha*, PHA, poly(hydroxybutyrate-*co*-hydroxyhexanoate), two-stage fed-batch, substrate-flexible, rapeseed oil, high-cell-density cultivation

## Abstract

Recent studies of the impact and dimension of plastic pollution have drawn the attention to finding more sustainable alternatives to fossil-based plastics. Microbially produced polyhydroxyalkanoates (PHAs) biopolymers are strong candidates to replace conventional plastic materials, due to their true biodegradability and versatile properties. However, widespread use of these polymers is still hindered by their high cost of production. In the present study, we target high yields of the PHA copolymer poly(hydroxybutyrate-*co*-hydroxyhexanoate) [P(HB-*co*-HHx)] using a substrate-flexible two-stage fed-batch approach for the cultivation of the recombinant *Cupriavidus necator* strain Re2058/pCB113. A more substrate-flexible process allows to cope with constant price fluctuations and discontinuous supply of feedstocks on the market. Utilizing fructose for biomass accumulation and rapeseed oil for polymer production resulted in a final biomass concentration of 124 g L^–1^ with a polymer content of 86 wt% holding 17 mol% of HHx. Productivities were further optimized by operating the biomass accumulation stage in a “drain and fill” modus where 10% of the culture broth was recycled for semi-continuous biomass accumulation, after transferring 90% to a second bioreactor for PHA production. This strategy succeeded in shortening process times rising productivity yields to ∼1.45 g L^–1^ h^–1^.

## Introduction

Out of the 407 million tons of plastics that are yearly produced worldwide over 35% are used as packaging materials, designed for immediate disposal after a single use (United Nations Environment Programm, 2018). Nevertheless, none of the commonly used plastics are biodegradable. As a result, they accumulate in landfills or the natural environment causing serious contamination problems (Jambeck et al., 2015; Geyer et al., 2017). In this scenario, a shift to sustainable plastic production that relies on renewable resources and does not threaten the environment is urgent. Key to the development of biologically derived biodegradable polymers is a class known as polyhydroxyalkanoates (PHAs). These linear polyesters are produced as water-insoluble storage polymers by a wide range of bacteria during nutrient limiting or stress conditions and the presence of excess carbon (Lenz and Marchessault, 2005; Dias et al., 2006; Obruca et al., 2020). Depending on the length of the side chain, PHA can be classified into *short*-, *medium-*, and *long-chain-length-* (*scl-*, *mcl-*, and *lcl-*)-polymers (Steinbüchel et al., 1992). PHAs exhibit thermoplastic and in the case of *mcl*-PHAs also elastomeric properties, similar to those of petroleum-based plastics. This makes PHAs suitable for a wide range of applications (Philip et al., 2007; Noda et al., 2010). After being disposed in environments with high microbial activity such as the soil, marine water or even in sewage sludge PHAs biodegrade easily within months (Ong et al., 2017; Meereboer et al., 2020).

Despite the considerable advantages of PHAs in terms of low environmental impact and highly tunable mechanical properties, production capacities for these polymer, although being one of the fastest growing amongst biopolymers, only account for around 1% of the global bioplastics extent (Vandi et al., 2018; European bioplastics, 2019). Barriers hindering PHA commercialization are mostly related to relatively higher costs, which makes it challenging to compete with low-priced petroleum-based plastics that are produced on a very large scale (Możejko-Ciesielska and Kiewisz, 2016; Sabapathy et al., 2020).

*Cupriavidus necator* (formerly *Ralstonia eutropha*) is the most studied organism for PHA production, mainly due to its ability to store the polymer up to 90% of its cell dry weight (CDW) under an ample spectrum of carbon sources and its diverse genetic modifiability (Pohlmann et al., 2006; Reinecke and Steinbüchel, 2008; Choi et al., 2020). The recombinant *C. necator* strain used in this study (Re2058/pCB113) was engineered to produce the poly(hydroxybutyrate-*co*-hydroxyhexanoate) [P(HB-*co*-HHx)] when fed with fatty acids containing feedstocks (Budde et al., 2011). Enhanced mechanical and thermal properties are depictured by this copolymer in comparison to poly(hydroxybutyrate) [P(HB)], which is typically produced by the wild-type strain or by this recombinant strain in the absence of an oleaginous substrate. The choice of a suitable carbon source is a main aspect in the optimization of PHA production, since it represents the highest cost driving factor, besides the downstream process (Koller et al., 2017). In this aspect, several inexpensive plant oils, predominantly palm oil and derivatives from the palm oil industry but also waste oleaginous feedstocks like waste cooking oils and animal by-products have been broadly used due to low prices and higher conversion rates to PHA in comparison to sugars (López-Cuellar et al., 2010; Riedel and Brigham, 2020). Nevertheless, it was pointed out that more flexible processes need to be developed in order to cope with constant price fluctuations and discontinuous supply of these feedstocks (Rodriguez-Perez et al., 2018). Developing a substrate-flexible process, where different substrates can be used for biomass accumulation and PHA production will reduce the dependency of a single feedstock. In addition, regarding bioprocess optimization to further decrease costs, only recently advanced cultivation strategies, like repeated batch and repeated fed-batch with bioreactors operated in a “drain and fill” modus were approached in order to achieve higher productivity yields and avoid the downtime between batches which results in high operation times (Koller, 2018).

In this study we report substrate-flexible two-stage fed-batch cultivations for the production of P(HB-*co*-HHx) using fructose during the biomass accumulation stage and rapeseed oil for polymer production. In order to achieve high space time yields (STY) a “drain and fill” modus is proposed for semi-continuous biomass production during the initial stage. Taking advantage of the high-cell-density achieved during the first stage, the second stage is run without previous sterilization of the in-series bioreactors.

## Materials and Methods

### Bacterial Strain, Preculture Conditions and Growth Media

Experiments were performed with the recombinant *C. necator* strain Re2058/pCB113, which produces the PHA copolymer P(HB-*co*-HHx), when grown on fatty acid containing feedstocks and polyhydroxybutyrate (PHB) when grown on fructose (Budde et al., 2011).

The seed train followed for the bioreactor cultivations consisted of two steps. First, a 125-mL Ultra Yield Flask^TM^ (Thomson Instrument Company, United States) containing 10 mL dextrose-free trypsic soy broth (TSB) medium and supplemented with 10 μg mL^–1^ gentamicin sulfate and 200 μg mL^–1^ kanamycin sulfate was inoculated with a single colony from an actively growing TSB agar plate, sealed with an AirOtop^TM^ membrane (Thomson Instrument Company, United States) and incubated for 17 h until reaching OD_583_ of 4–5.

Secondly, 1 mL of the pre-seed culture was used to inoculate a 500-mL DURAN^®^ baffled flask containing 100 mL phosphate buffered minimal medium that was sealed with an AirOtop membrane and incubated for around 26 h until OD_583_ of 4–5. The minimal media contained for each liter: 33.5 mL 1 M NaH_2_PO_4_, 64.5 mL 0.5 M Na_2_HPO_4_, 5.2 mL 0.5 M K_2_SO_4_, and 1 mL 1 M NaOH that were autoclaved together and then supplemented with the left sterile components: 20 mL 50% (w v^–1^) fructose, 20 mL 11.2% (w v^–1^) urea (or 20 mL 20% (w v^–1^) ammonium chloride for optimization of the biomass production stage), 10 mL 39 g L^–1^ MgSO_4_, 10 mL 6.2 g L^–1^ CaCl_2_, 1 mL 10 mg mL^–1^ gentamicin sulfate and 1 mL trace element solution. The trace element solution consisted of 15 g L^–1^ FeSO_4_⋅7H_2_O, 2.4 g L^–1^ MnSO_4_⋅H_2_O, 2.4 g L^–1^ ZnSO_4_⋅7H_2_O, and 0.48 g L^–1^ CuSO_4_⋅5H_2_O dissolved in 0.1 M hydrochloric acid. Incubation of the precultures was always performed at 30°C and 200 rpm in an orbital shaker (25 mm amplitude, INFORS HT Multitron Standard, Infors AG, Switzerland).

For bioreactor cultivations the unsterile components were sterilized *in situ* followed by addition of the sterile components to an initial volume of 0.5 L. Inoculation was performed with 20 mL of the pre-seed culture to an initial OD_583_ ≈ 0.2. After 24 h of cultivation MgSO_4_, CaCl_2_, K_2_SO_4_, and trace elements were added to initial concentrations in order to avoid limitation of these nutrients.

### Bioreactor Cultivation Conditions

For fed-batch cultivations 1-L Multifors parallel benchtop bioreactors (Infors AG, Switzerland) were used. The cultivation temperature was kept constant at 30°C and the pH maintained at 6.8 ± 0.1 through controlled addition of 1 M H_3_PO_4_ and 2 M NaOH (or 25% (v v^–1^) ammonia for pH-controlled feeding). Stirring was performed using two six-blade Rushton impellers. The initial stirring speed was set to 200 rpm, whereas the initial flow rate was set to 0.05 vvm. Via an automatized cascade, aeration was increased up to 0.5 vvm and later stirring was increased up to 1,500 rpm in order to prevent dissolved oxygen (DO) values from dropping below 40%. Foam was mechanically broken as described previously (Riedel et al., 2012). Additionally, silicon oil was added as antifoam when needed (maximum total amount added of 1 mL). The fed-batch cultivations were always performed in biological duplicates (two independent bioreactor cultivations) and consisted of two stages: a biomass accumulation stage and a PHA production stage.

#### Evaluation of Nitrogen Feeding in the Biomass Accumulation Stage

The biomass accumulation stage was conducted as indicated below with varying concentrations of nitrogen source in the feeding solution: without ammonium chloride, with 1% (w v^–1^) ammonium chloride and with 2% (w v^–1^) ammonium chloride. The cultivations were run for 48–50 h and the accurate concentration for ensuring availability of nitrogen throughout the complete biomass stage was tested. In the cultivations run afterward, ammonium chloride was replaced by corresponding concentrations or urea.

#### Two-Stage Fed-Batch Cultivation

##### Biomass accumulation stage

Initial batch phase until depletion of the 1% (w v^–1^) fructose present in the bioreactor followed by the automated start (triggered by sudden DO increase) of exponential feeding with 50% (w v^–1^) fructose and 0.56% (w v^–1^) urea at the specific growth rate μ_set_ according to

(1) $$\mu_{\text{set}}=0.75\cdot \mu_{\max}$$

(2) $$F\,\left(t\right)=F_{0}\cdot e^{\mu_{\text{set}}\cdot t}$$

The initial feed rate (L h^–1^) was calculated according to

(3) $$F_{0}=\,\frac{\mu_{\text{set}}}{Y_{X/S}\cdot S_{i}}\,\left(X_{0}\times V_{0}\right)$$

where Yxs the biomass/substrate yield (calculated from the batch phase), *S*_*i*_ the concentration of the carbon source in the feeding solution, and *X*_0_ and *V*_0_ the biomass concentration (calculated from a correlation between previous OD_583_ and CDW measured values) and bioreactor liquid volume at the end of the batch phase, respectively. During this stage the pH was controlled through addition of 25% ammonia in order to avoid nitrogen limitation. Feeding was performed until the measured OD_583_ exceeded 100 which marked the beginning of the second stage.

##### PHA production

PHA accumulation was triggered by nitrogen limitation. Therefore, the pH-control was switched from ammonia to 2 M NaOH. During this stage, a total amount of 135 g L^–1^ rapeseed oil were fed to the culture in a constant manner during the first 12 h. The culture was grown for further 32–36 h until the complete oil present in the bioreactor was consumed and the cells had achieved the highest PHA content.

#### Repeated Fed-Batch Cultivation With Semi-Continuous Biomass Accumulation

Three complete cycles of biomass accumulation and PHA production were run operating the biomass accumulation bioreactor in a “drain and fill” modus. The biomass accumulation stage was followed as indicated above until OD_583_ >100 and then 90% of the broth was transferred to a second bioreactor for PHA accumulation. To this end external periplasmic pumps were used. The main bioreactor was then refilled with sterile fresh media to a starting volume of 0.5 L and the biomass accumulation stage was repeated. Taking advantage of the high-cell-density achieved during the first stage, the second stage was run without previous sterilization of the bioreactors.

### Analytical Methods

For sampling, aliquots of 8 mL from bioreactor cultures were sampled in pre-weighted 15-mL tubes. The samples were centrifuged for 15 min and 4°C at 6,500 × *g*. The pellets were washed with 7 mL cold water (for samples during biomass accumulation) or with a mixture of 5 mL cold water and 2 mL cold hexane to remove residual oil (for samples during PHA production, when rapeseed oil was used as carbon source) and then dried at 80°C for CDW determination. The content and composition of PHA from dried cells was determined using a methanolysis protocol and gas chromatography as described previously (Bartels et al., 2020). The residual cell dry weight (RCDW) was defined as CDW minus the PHA content in g L^–1^. At every sampling point the OD_583_ of the culture broth was measured in duplicates, manually with a spectrophotometer (Ultraspec 3000, GE Healthcare, CT, United States; OD_583_-Photometer) and additionally with the automated pipetting system (Cedex Bio HT Analyzer^®^, Roche Diagnostics International AG, Switzerland; OD_583_-Cedex). Furthermore, 1 mL of the supernatant was filtered through an 0.2 μL PES syringe filter and used for fructose determination via HPLC-RID. Chromatography was run with 20 μL injection volume at 80°C for 62 min on an Agilent Hi-Plex Ca column. The eluent was DI H_2_O with an 0.6 mL min^–1^ flux. Unfiltered supernatant was measured with the Cedex Bio HT Analyzer to assess consumption of NH_3_, Mg^2+^, PO_4_^3–^, and Ca^2+^.

## Results

### Evaluation of Nitrogen Feeding in the Biomass Accumulation Stage

**FIGURE 1:**
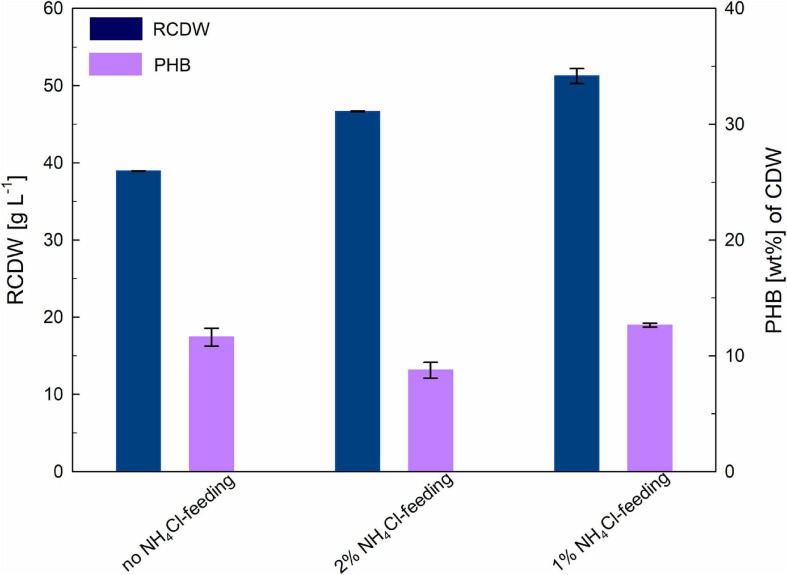
Optimization of biomass production stage: the achieved residual cell dry weight (RCDW; g L**^–^**^1^) and PHB content (wt%) after 48–50 h of cultivation is indicated for every condition. Error bars indicate standard deviation from two independent bioreactor cultivations.

For an initial evaluation of the optimal conditions for ensuring nitrogen availability throughout the complete biomass accumulation stage, three different strategies were evaluated; providing nitrogen only through the pH-controlled feeding with 25% (v v^–1^) ammonia, adding also 1% (w v^–1^) ammonium chloride to the fructose feeding solution and adding 2% (w v^–1^) ammonium chloride to the fructose feeding solution. It is important to mention that after nitrogen limitation the bacteria immediately cease growth and engage polymer accumulation, this limiting final biomass yields. Figure 1 shows an overview of the final RCDW and PHB values yielded by each cultivation after 48–50 h.

When no nitrogen source was added to the fructose feeding solution (see Supplementary Figure 1), NH_3_ concentrations measured showed a notorious decrease after the beginning of the fed-batch phase (from 120 to 3 mM). The inability of the pH-controlled nitrogen feeding to keep up with the nitrogen consumption of the culture triggered a premature accumulation of PHB, which lowered the final biomass yield of the cultivation. After 48 h a RCDW of 38 g L^–1^ with a PHB content of 12 wt% had been reached.

In order to circumvent nitrogen limitation during the biomass accumulation stage, addition of two different concentrations of ammonium chloride to the fructose feeding solution was tested (see Supplementary Figure 2). When 1% (w v^–1^) ammonium chloride was added, the feeding succeeded in compensating the consumption of the cells; although NH_3_ concentration slightly dropped during the fed-batch phase it never went below 80 mM. This cultivation showed the highest biomass yield with 51 g L^–1^ RCDW and a PHB content of 12 wt% after 50 h of cultivation. Regarding the feeding with 2% (w v^–1^) ammonium chloride, it could be observed that during the fed-batch phase NH_3_ quickly accumulated in the bioreactor doubling the initial concentrations. Final titers after 50 h of cultivation showed a RCDW of 45 g L^–1^ with a PHB content of 11 wt% of CDW.

Exponential feeding with 50% (w v^–1^) fructose and 1% (w v^–1^) ammonium chloride in addition to the pH-controlled feeding with 25% (v v^–1^) ammonia was chosen as the best strategy to ensure nitrogen availability throughout the complete biomass production stage. In the following cultivations the fructose feeding was supplemented with 0.56% (w v^–1^) urea (corresponding to 1% (w v^–1^) ammonium chloride (187 mM NH_4_^+^).

### Two-Stage Fed-Batch Cultivation

**FIGURE 2:**
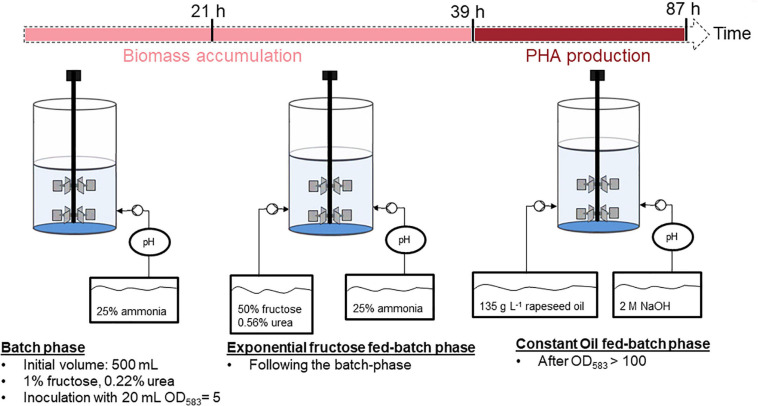
Overview of the substrate-flexible two-stage fed-batch strategy.

An overview of the substrate-flexible two-stage fed-batch strategy for P(HB-*co*-HHx) production with fructose feeding for the biomass accumulation stage and rapeseed oil feeding for polymer production is presented in Figure 2.

**FIGURE 3:**
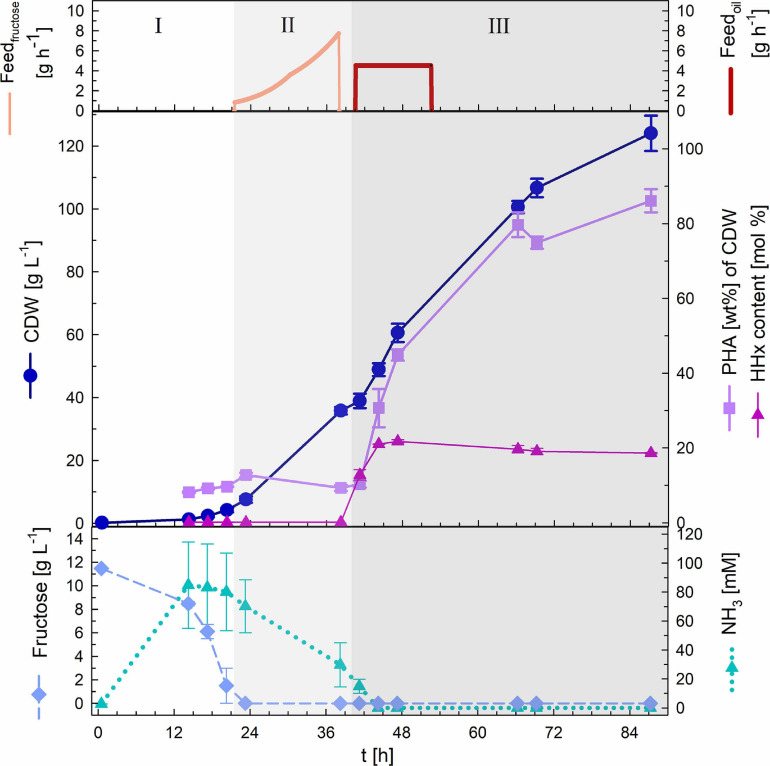
Substrate-flexible two-stage fed-batch cultivation. Upper graph: fructose feed (g h^–1^) and oil feed (g h^–1^). Middle graph: cell dry weight (CDW; g L^–1^), PHA content (wt%) and HHx content (mol%). Lower graph: fructose (g L^–1^) and NH_3_ (mM) concentrations. Biomass accumulation stage: batch phase (I) and exponential fructose fed-batch phase (II). PHA production stage: constant rapeseed oil fed-batch phase (III). Error bars indicate standard deviation from two independent bioreactor cultivations.

The cultivation was performed in biological duplicates (two independent bioreactor cultivations) and the results are depictured below (Figure 3). The batch phase lasted ∼21 h and showed a maximal growth rate of 0.22 h^–1^ and an average yield of 0.44 (g biomass/g fructose). After depletion of the 1% (w v^–1^) fructose present in the bioreactor exponential feeding with 50% (w v^–1^) fructose and 0.56% (w v^–1^) urea at a *μ*_set_ of 0.15 h^–1^ was performed for 18 h. During this period around 100 mL of feeding solution was provided to the culture. Fructose remained limiting during the complete fed-batch phase with HPLC measurements showing undetectable concentrations. Furthermore, ammonia measurements showed permanent availability of nitrogen with a minimal concentration of 30 mM NH_3_. It is important to mention that the availability of the nitrogen source, urea, was detected only indirectly. Urea is taken up by the bacteria and hydrolyzed by the cytoplasmic urease enzyme complex, leading to one CO_2_ and two ammonium molecules (Beckers et al., 2004). In this study, only measurements of NH_3_ were performed. After 39 h of cultivation 36 g L^–1^ CDW with a PHB content of 9.3 wt% of CDW had been attained. After measuring optical densities greater than OD_583_ = 100 the second stage of the cultivation was started triggering nitrogen limitation by changing the pH-control to NaOH and feeding with rapeseed oil to enable the incorporation of the *mcl*-HHx-monomers into the PHA polymer. During the first 12 h of this stage a total amount of 135 g L^–1^ rapeseed oil were fed at a constant feeding rate. Nitrogen had been depleted within 6 h and after a total period of 87 h a final CDW of 124 g L^–1^ and a P(HB-*co*-HHx) content of 86.1 wt% of CDW with an HHx level of 16.9 mol% had been achieved. The P(HB-*co*-HHx) STY was calculated to be 1.22 g L^–1^ h^–1^.

### Repeated Fed-Batch Cultivation With Semi-Continuous Biomass Accumulation

While the two-stage fed-batch cultivation aimed to evaluate the feasibility of achieving high P(HB-*co*-HHx) titers using fructose for the biomass accumulation stage and rapeseed oil for polymer production and served as a reference for this process, the repeated fed-batch cultivations performed later in the study and presented here engaged with the goal of developing a more time-effective process with optimized STY.

**FIGURE 4:**
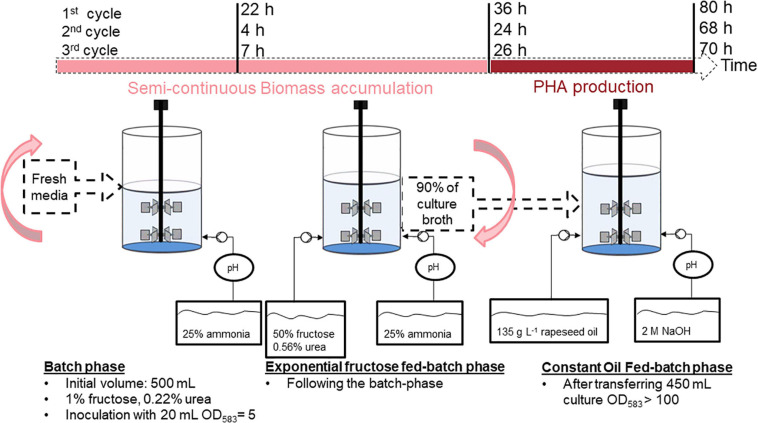
Overview of the substrate-flexible two-stage repeated fed-batch cultivation strategy with semi-continuous biomass accumulation.

To this end, a strategy consisting of a repeated fed-batch based on a “drain and fill” operation modus for semi-continuous biomass accumulation was developed for the first stage of the process. 90% of the high-cell-density culture delivered from the first stage was then transferred into a second bioreactor, which needed no previous sterilization and served for polymer accumulation in the second stage of the process. Three complete cycles of biomass accumulation and polymer production were conducted over a total period of 1 week. An overview of the chosen strategy, including the times needed for each cycle is provided in Figure 4. A detailed sketch of the cultivations is provided in Supplementary Figure 3.

#### Biomass Accumulation

**FIGURE 5:**
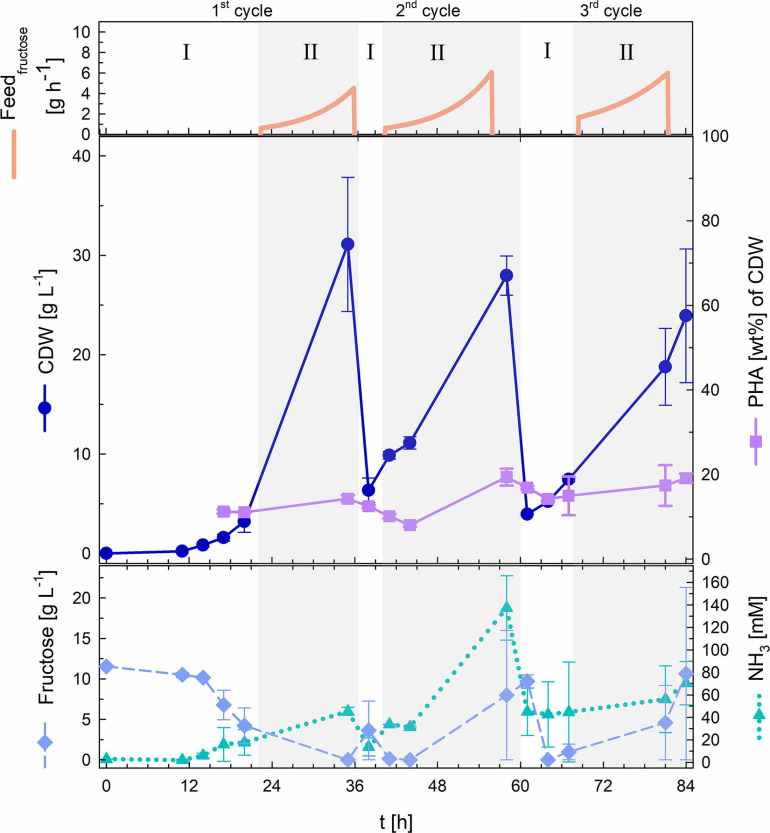
Repeated fed-batch cultivation with semi-continuous biomass accumulation. Upper graph: fructose feed (g h**^–^**^1^). Middle graph: cell dry weight (CDW; g L**^–^**^1^) and PHA content (wt%). Lower graph: fructose (g L**^–^**^1^) and NH_3_ (mM) concentrations. Three cycles of biomass accumulation: batch phase (I) and exponential fructose fed-batch phase (II). Error bars indicate standard deviation from two independent bioreactor cultivations.

Figure 5 delivers key information gained from the repeated fed-batch strategy that was applied for the three cycles of semi-continuous biomass accumulation. The first cycle of biomass accumulation (0–36 h) involved an initial batch phase of 22 h that was characterized by a long lag phase of around 11 h after which the culture showed a *μ*_max_ of 0.23 h^–1^. After fructose depletion, exponential feeding at a *μ*_set_ of 0.15 h^–1^ was applied for 14 h. During this first fed-batch phase fructose remained at undetectable concentrations and NH_3_ measurement showed permanent availability of nitrogen with a maximal value of 45 mM. At the end of the first biomass accumulation cycle 31.1 g L^–1^ CDW with a PHB content of 14.3 wt% had been reached. After this timepoint 90% of the high-cell-density culture broth was withdrawn and the left 60 mL were recycled for the next cycle of biomass accumulation that was started after refilling the bioreactor with fresh media to an initial volume of 0.5 L.

The second cycle of biomass accumulation (36.5–60.5 h) showed a comparably much shorter batch phase of only 4 h (with no detectable lag phase) within which the complete 10 g L^–1^ fructose provided in the fresh media had been consumed. Feeding was then performed for 20 h at again, a *μ*_set_ of 0.15 h^–1^. At the end of the second fed-batch phase fructose and NH_3_ had accumulated in the bioreactors reaching 8 g L^–1^ and 137 mM respectively. Biomass measurements showed final values of 28 g L^–1^ with a PHB content of 19.4 wt%.

For the third and last cycle of biomass accumulation (61–84 h), 60 mL of culture broth from the second cycle were again recycled. The third batch phase lasted 7 h and was followed by 19 h of exponential fed-batch phase. At the end of the fed-batch phase fructose and NH_3_ had accumulated in the bioreactors (21 g L^–1^ fructose and 90 mM NH_3_). Final values of the third biomass accumulation cycle showed 24 g L^–1^ CDW and a PHB content of 19.1 wt%.

Recycling 10% of the biomass at the end of each cycle reduced batch times needed for initial biomass accumulation from 22 h (1^st^ cycle) to 4–7 h (2^nd^ and 3^rd^ cycle).

#### PHA Production

**FIGURE 6:**
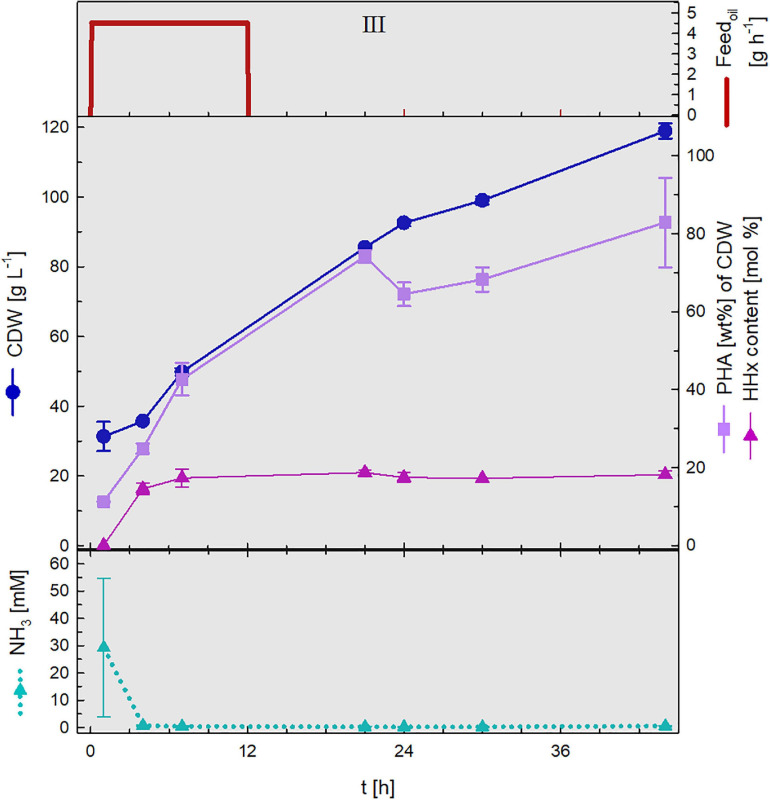
PHA production stage. Upper graph: oil feed (g h**^–^**^1^). Middle graph: cell dry weight (CDW, g L**^–^**^1^), PHA content (wt%) and HHx content (mol%). Lower graph: NH_3_ (mM) concentrations. Constant rapeseed oil fed-batch phase (III) of the 1^st^ cycle. Error bars indicate standard deviation from two independent bioreactor cultivations.

During the second stage of the cultivation, which was conducted in a second bioreactor, polymer accumulation was triggered by nitrogen limitation. Nitrogen source was no longer provided and feeding with rapeseed oil permitted the incorporation of *mcl*-monomers into the PHA polymer. Figure 6 illustrates such stage, in this case, the data presented was gained from the first cycle of polymer production.

The culture broth withdrawn from the first biomass accumulation stage that presented relatively low NH_3_ concentrations (30 mM) showed nitrogen depletion after 4 h after which time P(HB-*co*-HHx) enrichment was set off reaching maximal concentrations of 82.9 wt% with an HHx level of 18.1 mol%. At the end of the first cycle 119 g L^–1^ CDW had been attained.

In order to guarantee high P(HB-*co*-HHx) production during the second stage of the process, it was mandatory to withdraw from the first stage a culture broth that contained no excess nitrogen concentrations (see Table 1). It was observed that NH_3_ concentrations that did not exceed 45 mM (see two-stage fed-batch and repeated fed-batch 1^st^ cycle) were consumed within the first few hours of the second stage after which still enough carbon source was fed to trigger the accumulation of high PHA concentrations. When overfeeding during the first stage of the process resulted in ammonia accumulation in the bioreactor (see repeated fed-batch 2^nd^ and 3^rd^ cycle) polymer production during the second stage was strongly hindered.

**TABLE 1:** Comparison of growth and PHA production during all cultivations.

	Biomass accumulation stage				PHA production stage			Overall
	CDW (g L^–1^)	PHB (wt%)	NH_3_ (mM)	Duration (h)	CDW (g L^–1^)	P(HB-*co*-HHx) (wt%)	HHx (mol%)	STY (g L^–1^ h^–1^)
Two-stage fed-batch	35.8 ± 1.2	9.3 ± 0.8	29.7 ± 15.3	39	124.0 ± 5.6	86.1 ± 3.1	16.9 ± 0.2	1.22
Repeated fed-batch 1^st^ cycle	31.1 ± 6.7	14.3 ± 0.8	45.4 ± 3.6	36	118.9 ± 2.1	82.9 ± 11.5	18.1 ± 0.9	1.23
Repeated fed-batch 2^nd^ cycle	27.9 ± 1.9	19.4 ± 2.0	137.3 ± 28.6	24	35.8 ± 10.1	33.9 ± 29.2	20.6 ± 2.4	0.18*
Repeated fed-batch 3^rd^ cycle	23.9 ± 6.7	19.1 ± 1.1	70.4 ± 19.3	26	53.6 ± 18.7	56.3 ± 18.4	13.5 ± 2.3	0.43*

*CDW and PHA concentrations, including HHx content for the production stage, where P(HB-*co*-HHx) was synthesized, are displayed. In addition, final PHA STYs are shown for each run. NH_3_ accumulation at the end of the biomass accumulation stage is presented in order to understand the low polymer yields(^∗^) attained in the 2^*nd*^ and the 3^*rd*^ cycle of the repeated fed-batch. In addition, the duration of the biomass accumulation stage shows the advantage of the repeated fed-batch approach. Measurements represent means from duplicate cultivations ± are indicating minimum and maximum values.*

### Comparison of Growth and PHA Production During All Cultivations

First, the biomass accumulation stage was optimized to RCDW > 50 g L^–1^ by ensuring nitrogen availability throughout the complete stage, thus retarding polymer production (Figure 1). It was determined, that supplementing the fructose feeding with 187 mM ammonium in addition to the pH-controlled feeding with 25% (v v^–1^) ammonia was the best strategy to avoid premature polymer enrichment. Moving forward, after reaching high biomass concentrations, this was followed by a polymer production stage with rapeseed oil feeding. A CDW of 124 g L^–1^ and a P(HB-*co*-HHx) content of 86.1 wt% with an HHx level of 16.9 mol% after 87 h with a STY of 1.22 g L^–1^ h^–1^ was achieved (Table 1 and Figure 3). Next, a repeated two-stage fed-batch cultivation with semi-continuous biomass accumulation was developed. By variation of the biomass accumulation stage into cyclic mode (“drain and fill”), the need of new precultures and the non-productive time of cleaning and setting up the bioreactor for the initiation of a fresh cultivation could be avoided. The culture lag phase, of around 11 h, observed at the beginning of the batch phase in the first approach was significantly reduced by recycling 10% of the high-cell-density biomass of each cycle (Figure 5). This served as inoculum when the bioreactor was refilled with fresh media whereas 90% of the culture broth was transferred into a second bioreactor for polymer accumulation. By this, a time reduction of the batch phase from 22 h to 4–7 h was observed in the cyclic approach (Figure 5). Taking only the reduction of the batch phase into consideration the STY of the process could be increased by 20% to a potential STY of ∼1.45 g L^–1^ h^–1^.

## Discussion

The purpose of our study was to develop a substrate-flexible two-stage fed-batch process where fructose was only utilized for cell growth and P(HB-*co*-HHx) accumulation was only triggered in combination with rapeseed oil feeding. The P(HB-*co*-HHx) yields from rapeseed oil where between 0.70–0.76 g g^–1^ with HHx contents of around 17 mol%. This strongly correlates to results obtained in high-cell-density cultivations with the same strain, where only plant oil was used as the carbon source (Riedel et al., 2012; Madison et al., 2014). Previously published studies with *C. necator* Re2058/pCB113 showed a decrement of the HHx content when using mixtures of sugars and plant oils during the whole cultivation (Murugan et al., 2016, 2017; Purama et al., 2018). This effect also occurred during the PHA production phase of the 2^nd^ and 3^rd^ cycle, where fructose was still present due to overfeeding of the biomass accumulation stage (Figure 5 and Table 1). Early PHA production with *C. necator* Re2058/pCB113 was reported previously and is attributed to the PHA production genes being located on an overexpression plasmid (Budde et al., 2011). However, interestingly, in this study the HHx content did not decrease during the 1^st^ cycle and the two-stage fed-batch (without fructose overfeeding), although the strain had already accumulated around 20 wt% PHB during the biomass accumulation from fructose (Figures 3, 5Table 1). Therefore, the method presented herein is able to increase substrate flexibility without affecting final yields and polymer composition. However, the effect on the thermal, physical, mechanical features and the molecular weight has to be investigated in further studies. The weight average molecular weight of P(HB-*co*-HHx) produced with *C. necator* Re2058/pCB113 has been reported to be in the range of 3.0–6.0 × 10^5^ Da when grown on plant oils as sole carbon source (Riedel et al., 2012; Zainab-L et al., 2018). Using mixtures of palm oil and fructose or seed oil and molasses as carbon sources for *C. necator* Re2058/pCB113 cultivations (total CDW < 10 g L^–1^), lead to average molecular weights in the range of 5.5–8.3 × 10^5^ with controllable HHx-contents between 4–28 mol% HHx (Murugan et al., 2017; Purama et al., 2018).

Efforts in optimizing the initial growth stage with fructose feeding succeeded in rising the RCDW up to 50 g L^–1^ before the main polymer production phase (Figure 1). To avoid premature nitrogen limitation, urea was chosen as an additional nitrogen source beside pH-controlled ammonia feeding. We took notice, that during the 2^nd^ and 3^rd^ cycle of biomass accumulation overfeeding led to accumulation of NH_3_ in the bioreactors (Table 1 and Figure 5). Recent studies have suggested that high NH_3_ levels may trigger a stress response, involving the formation of (p)ppGpp alarmone, that could trigger preliminary PHB accumulation without nutrient starvation (Gutschmann et al., 2019). The high concentrations of NH_3_ affected the overall yield of this cycles that were characterized by comparably lower CDWs and polymer accumulation. In the future, it could be considered to apply urea feeding only for the 1^st^ cycle of cell growth which as this cycle showed to be considerably longer and thus is more prone to undergo nutrient limitations.

The very high STY of 1.22 g PHA L^–1^ h^–1^ accomplished in this study is comparable to other published high-cell-density cultivations from plant oils (Kahar et al., 2004; Obruca et al., 2010; Riedel et al., 2012; Arikawa and Matsumoto, 2016; Gutschmann et al., 2019). Productivities were further optimized by adopting an advanced cultivation strategy (“drain and fill”) for semi-continuous biomass production in the first stage of the process. Doing these, initial long lag phases (Figures 3, 5) could be avoided reducing the overall process time in ∼20%, showing the potential to increase the STY to ∼1.45 g P(HB-*co*-HHx) L^–1^ h^–1^. However, it is important to note that this fed-batch productivity is reported on a timescale from inoculation to harvest. To be able to compare values from the fed-batch approach and the repeated fed-batch approach with semi-continuous biomass accumulation, productivity should be amortized over time from one harvest to the next, considering the downtime for cleaning, setup, sterilization, and preparation of the inoculum for the subsequent cultivation (Blunt et al., 2018). In our study the P(HB-*co*-HHx) production was triggered under unsterile conditions in separate bioreactors. This could avoid sterilization costs, a major price factor in biotechnological processes (Wang et al., 2014).

Only recently repeated batch and fed-batch strategies have been reported for optimized PHA production (Singhaboot and Kaewkannetra, 2015; Gahlawat et al., 2017). To the best of our knowledge, to date, this is the first report on a repeated fed-batch strategy where the “drain and fill” protocol is used for semi-continuous biomass accumulation whereas high polymer concentrations, of over 100 g L^–1^ P(HB-*co*-HHx) are attained in-series bioreactors. According to Ienczak et al. (2013), even a total PHA production of ∼200 g L^–1^ would be possible based on the achieved high RCDW (∼50 g L^–1^) in this study.

## Conclusion

As a conclusion, the data presented herein describes the production of the PHA copolymer P(HB-*co*-HHx) utilizing fructose and rapeseed oil as feedstocks in different stages of the process. Optimized polymer productivities of ∼1.45 g L^–1^ h^–1^ with a total PHA production up to 100 g L^–1^ were reached through a repeated fed-batch process with semi-continuous biomass accumulation. The new method described in this study not only reduced the process times related to long lag-phases at the beginning of each batch (time reduction of ∼20%) but also circumvented the need of laborious pre-seed cultures. Taking advantage of the high-cell-densities achieved before triggering polymer accumulation (RCDW > 30 g L^–1^) performing the second stage in unsterile bioreactors allowed the sparing of sterilization costs and time. Results suggest that applying the method presented here could contribute to reduce production costs and, in this way, accelerate the commercialization of a sustainable PHA-bioplastic.

## Data Availability Statement

The original contributions presented in the study are included in the article/Supplementary Material, further inquiries can be directed to the corresponding author/s.

## Author Contributions

SR contributed to the conception and design of the study. LS and SW carried out the experiments and analysis of the data. LS and SR prepared the first draft of the manuscript. SR and PN were responsible for the project administration and funding acquisition. All authors contributed to the manuscript revision, read and approved the submitted version.

## Conflict of Interest

The authors declare that the research was conducted in the absence of any commercial or financial relationships that could be construed as a potential conflict of interest.
