# Supplementary material for: Substrate-Flexible Two-Stage Fed-Batch Cultivations for the Production of the PHA Copolymer P(HB-co-HHx) With Cupriavidus necator Re2058/pCB113
Source: Front Bioeng Biotechnol. 2021 Mar 22;9:623890. doi: 10.3389/fbioe.2021.623890 (PMC8020817; doi:10.3389/fbioe.2021.623890)
Supplement: Supplementary file 1 [file Data_Sheet_1.pdf]

frontiers

Supplementary Material

Substrate-flexible two-stage fed-batch cultivations for the production of the PHA Copolymer P(HB-*co*-HHx) with *Cupriavidus necator* Re2058/pCB113

Lara Santolin[1], Saskia Waldburger[1], Peter Neubauer[1], Sebastian L. Riedel[1*]

[1]Technische Universität Berlin, Institute of Biotechnology, Chair of Bioprocess Engineering, Berlin, Germany

*** Correspondence:**
Sebastian L. Riedel
riedel@tu-berlin.de

1 Supplementary Figures

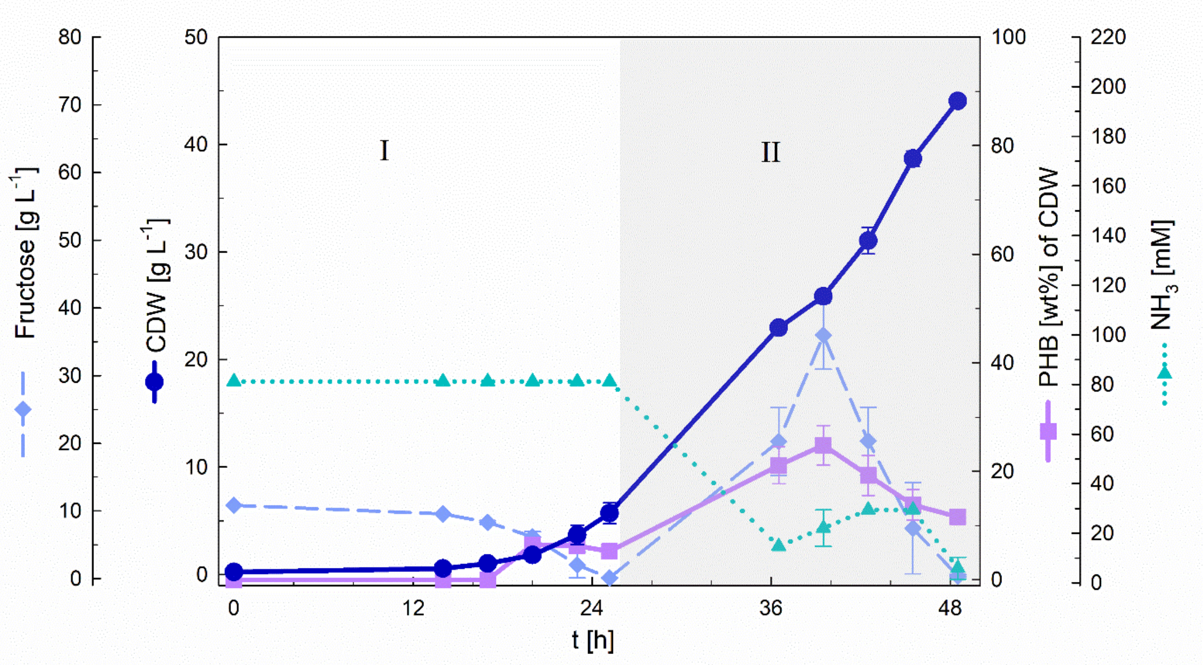

Supplementary Figure 1. Biomass accumulation stage without ammonium chloride feeding. Fructose (g L^{-1}), cell dry weight (CDW, g L^{-1}), PHB content (wt%) and NH_3 (mM) concentrations. Error bars indicate standard deviation between biological duplicates. Batch phase (I), fed-batch phase (II).

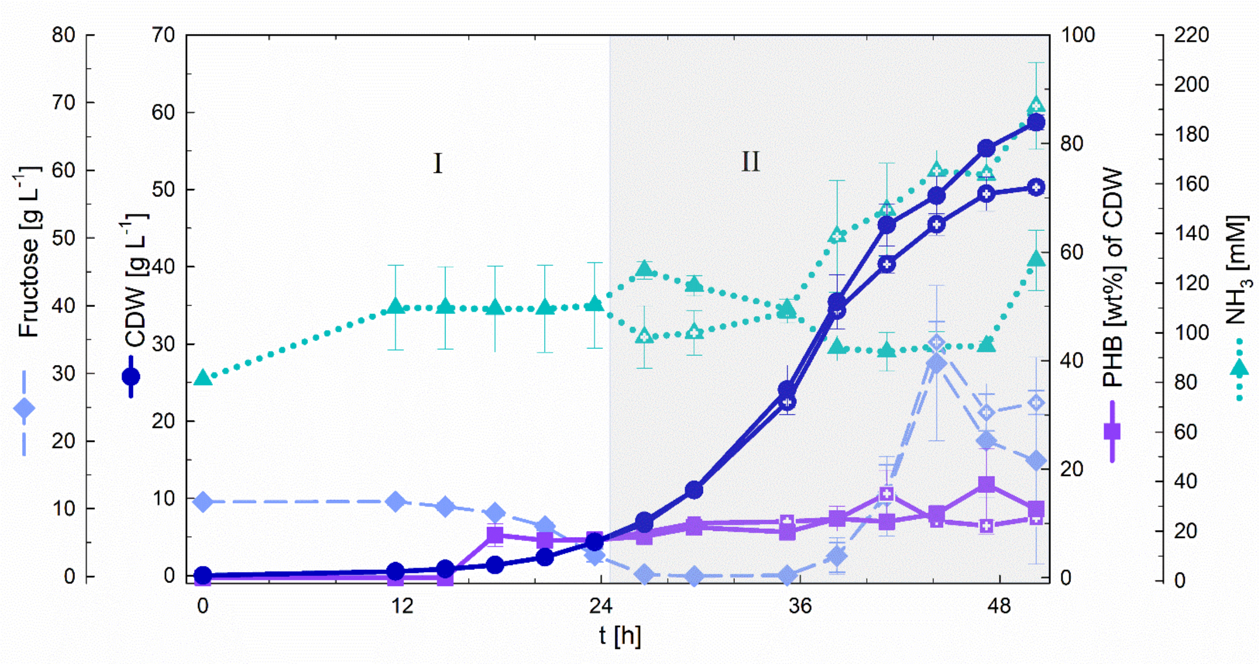

Supplementary Figure 2. Biomass accumulation stage with ammonium chloride feeding. Fructose (g L^{-1}), cell dry weight (CDW, g L^{-1}), PHB content (wt%) and NH_3 (mM) concentrations. Data from feeding with 1% (w v^{-1}) NH_4Cl is represented with empty symbols, data from feeding with 2% (w v^{-1}) NH_4Cl by dotted symbols. For the batch phase (I) the error bars indicate standard deviation between all four biological replicates, for the fed-batch phase (II) the error bars indicate standard deviation between biological duplicates.

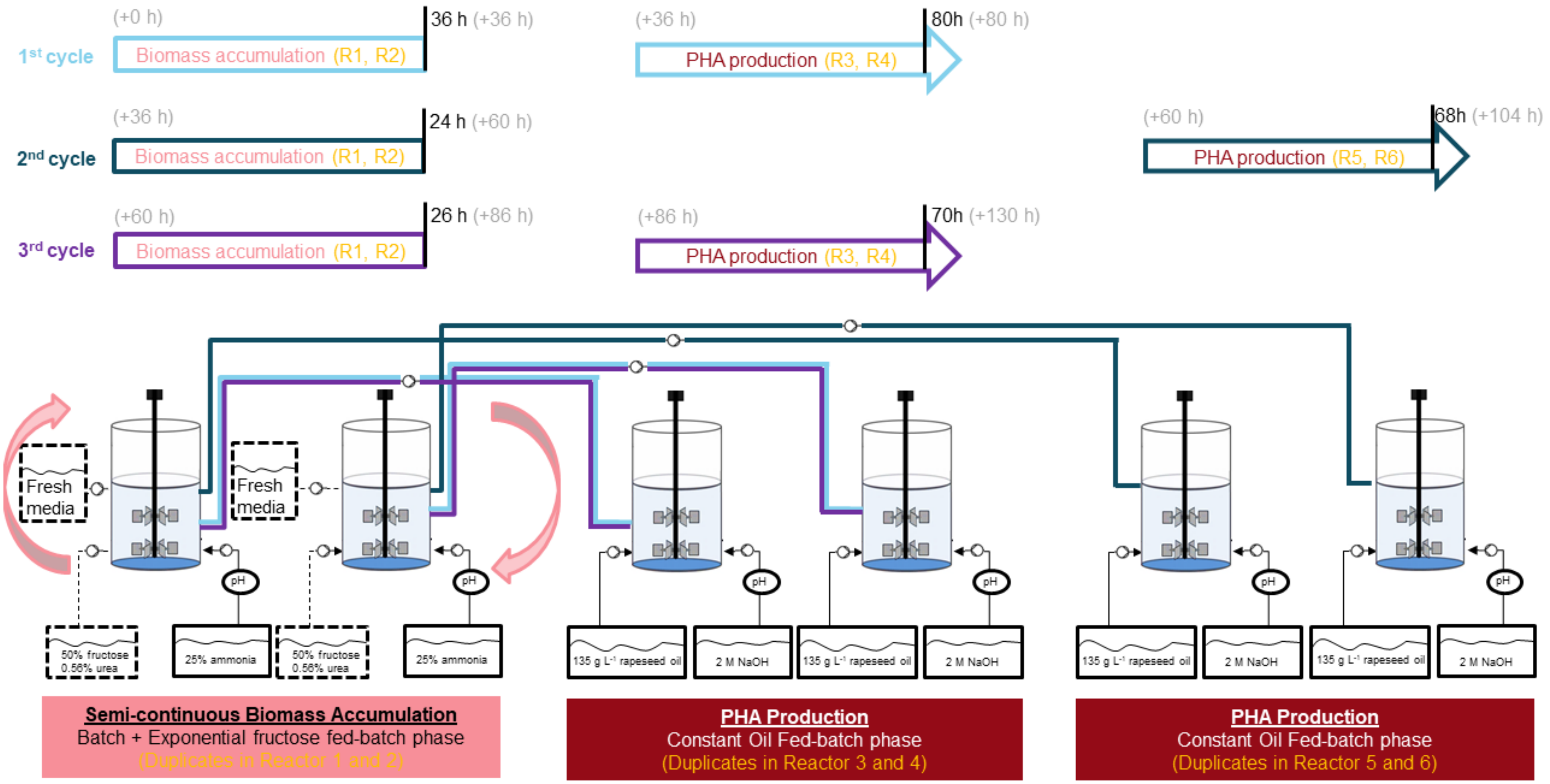

Supplementary Figure 3. Detailed overview of the three cycles of two-stage repeated fed-batch cultivation with semi-continuous biomass accumulation. 6 bioreactors were used for the study using always two for duplicates (R1 and R2, R3 and R4, R5 and R6). The biomass accumulation stage, consisting of an initial batch phase followed by an exponential fed-batch phase with fructose was performed in R1 and R2. After each cycle of biomass accumulation, 90% of the culture broth was transferred to a second bioreactor (R3 and R4 in the 1st cycle (light blue), R5 and R6 in the 2nd cycle (dark grey), R3 and R4 in the 3rd cycle (violet)). The left culture broth in R1 and R2 was recycled filling

up the bioreactors with new media for the next cycle. The hours indicated in grey show the overall timescale, starting at the inoculation of the 1st cycle. The absolute times needed for each cycle are shown in black.
